# Rapid and Sensitive Detection of Rotavirus Molecular Signatures Using Surface Enhanced Raman Spectroscopy

**DOI:** 10.1371/journal.pone.0010222

**Published:** 2010-04-19

**Authors:** Jeremy D. Driskell, Yu Zhu, Carl D. Kirkwood, Yiping Zhao, Richard A. Dluhy, Ralph A. Tripp

**Affiliations:** 1 Department of Infectious Diseases, University of Georgia, Athens, Georgia, United States of America; 2 Department of Physics, University of Georgia, Athens, Georgia, United States of America; 3 Department of Chemistry, University of Georgia, Athens, Georgia, United States of America; 4 Enteric Virus Group, Murdoch Children's Research Institute, The Royal Children's Hospital, Parkville, Victoria, Australia; Erasmus Medical Center, Netherlands

## Abstract

Human enteric virus infections range from gastroenteritis to life threatening diseases such as myocarditis and aseptic meningitis. Rotavirus is one of the most common enteric agents and mortality associated with infection can be very significant in developing countries. Most enteric viruses produce diseases that are not distinct from other pathogens, and current diagnostics is limited in breadth and sensitivity required to advance virus detection schemes for disease intervention strategies. A spectroscopic assay based on surface enhanced Raman scattering (SERS) has been developed for rapid and sensitive detection of rotavirus. The SERS method relies on the fabrication of silver nanorod array substrates that are extremely SERS-active allowing for direct structural characterization of viruses. SERS spectra for eight rotavirus strains were analyzed to qualitatively identify rotaviruses and to classify each according to G and P genotype and strain with >96% accuracy, and a quantitative model based on partial least squares regression analysis was evaluated. This novel SERS-based virus detection method shows that SERS can be used to identify spectral fingerprints of human rotaviruses, and suggests that this detection method can be used for pathogen detection central to human health care.

## Introduction

Group A rotaviruses are the leading cause of acute severe gastroenteritis in infants and young children worldwide with approximately 130 million children infected each year. This accounts for approximately 1/3 of all hospital admissions each year for diarrheal disease and is estimated to be responsible for over 500,000 deaths, 2 million hospitalizations, and 25 million clinic visits each year [1]. Rotaviruses are extremely infectious and pose a significant burden on health care worldwide, thus surveillance methods are necessary to track outbreaks of current and emerging strains, as well as aid in the development of vaccine and disease intervention strategies.

Rotaviruses are non-enveloped icosahedral particles containing 11 segments of dsRNA [2], which are further categorized based on three layers, i.e. an inner core, an inner capsid and an outer capsid [3]. The inner capsid protein, VP6, is conserved among all group A rotaviruses [4]. The outer capsid consists of two proteins, VP7 and VP4, that are the major neutralizing antigens with each independently segregating. Rotavirus has a dual typing system based on the 2 outer capsid proteins, classification based on VP7 are termed G genotypes and VP4 are termed P genotypes. At present, 11 of 15 G types, i.e. VP7 variants, and 12 of 26 P types, i.e. VP4 variants, are known to infect humans [5]. On a global basis, most severe infections are caused by five G types (G1–G4 and G9) and three P types (P1A[8], P1B[4], and P2A[6]), although considerable epidemiological differences exist in some areas especially in tropical countries [5], [6].

Commercial immunochromatographic assays and enzyme immunoassays are available for routine laboratory diagnosis of rotavirus in a clinical setting. These assays capitalizes on the conserved nature of VP6 among all group A rotaviruses; however, these tests provide no information on the genotypes, i.e., G and P types, which is essential for monitoring epidemics, identifying novel strains, and in controlling disease. Typing of rotavirus strains is achieved using genotype specific monoclonal antibodies (mAbs) in an ELISA assay, and by hemi-nested multiplex RT-PCR [5], [7]. These methods are labor intensive, reliant on species-specific reagents (e.g. mAbs and genotyping primers) and particularly for PCR, amplification of the analyte for detection. There is an unmet need for a rapid, sensitive, and specific means of detecting and differentiating rotavirus strains.

Surface enhanced Raman spectroscopy (SERS) provides the ability to rapidly detect analytes with chemical specificity intrinsic to vibrational spectroscopy and is emerging as an important tool in bioanalytical applications including identification and classification of pathogenic organisms [8], [9], [10], [11]. Historically, Fourier transform infrared spectroscopy (FTIR) and Raman scattering have been explored as vibrational spectroscopic techniques for the detection and differentiation of infectious agents [12], [13], [14], [15], [16], [17], [18], [19], [20], [21]. These methods provide detailed information regarding the chemical composition of pathogens which serve as fingerprints for detection and identification. While each has achieved success in whole-organism fingerprinting, it has been found that each suffers from inherent limitations. For example, FTIR is limited by interference from water; while conversely, Raman spectroscopy, while providing spatial resolution and resistance to water, is severely limited by low scattering cross sections which translate to weak signals for detection. The Raman signal of a sample, e.g., pathogen, can be significantly enhanced via adsorption to a metallic nanostructured surface in a technique referred to as surface-enhanced Raman spectroscopy (SERS). The signal amplification results from an increased electromagnetic field experienced by the molecules in close proximity to the metal surface. Briefly, the appropriate choice of laser excitation frequency excites the conduction electrons in a metal surface with requisite nanometric size to collectively oscillate generating a localized and intensified electromagnetic field [22]. The enhancement effect is system dependent, e.g., substrate and analyte, with typical enhancements of 10^4^ to 10^14^ with respect to normal Raman intensities. Importantly, SERS retains all of the benefits of normal Raman spectroscopy while providing a markedly improved sensitivity, and as a result, SERS has advanced as the spectroscopic tool of choice for whole-organism fingerprinting [8], [9], [10], [11], [23], [24], [25], [26], [27], [28], [29], [30].

The majority of SERS-based detection assays have been developed for bacteria [9], [24], [25], [26], [27], [28], [29], [30], [31], although SERS detection of viral pathogens is emerging [8], [10], [11], [23], [32], [33]. Despite the improved detection features offered by SERS, several bacterial detection studies have reported different spectra for the same organism. For example, SERS spectra have been published for both *Bacillus subtilis* and *Escherichia coli*; however in each report an incongruent spectral fingerprint was indicated [25], [26], [29], [30]. The apparent discrepancies can be attributed to a critical component in the SERS assay, i.e., the substrate. The SERS spectrum is dependent on the Raman signal enhancing substrate, thus a reliable method for fabricating reproducible substrates is critical for SERS-based assays. This aspect has prevented the widespread use of SERS-based detection assays. We have recently addressed this challenge with the development of a silver nanorod array substrate prepared via oblique angle vapor deposition (OAD). The OAD process produces high aspect ratio silver nanorods yielding a SERS enhancement factor of >10^8^ with less than 15% variation in SERS intensity from batch-to-batch [34].

The method of data analysis is also a critical aspect of any diagnostic assay, particularly for vibrational spectroscopy. It is important to analyze the entire spectrum, or use specialized feature selection algorithms, since discrete patterns of multiple bands, rather than a single peak, are important for identification. Principal component analysis (PCA) is the most frequently employed multivariate technique used to reduce the dimensionality of the spectral dataset, reduce noise, and maximize total spectral variance among spectral fingerprints for each infectious agent [35], [36]. PCA is used to evaluate the reproducibility and specificity of the spectroscopic technique, but ultimately functions to cluster similar spectra into groups for classification. PCA has been successfully applied to spectroscopic assays of bacteria and viruses. A slightly more sophisticated category of multivariate analysis is supervised methods, whereby a calibration dataset of known identity is required to build a classification model. These methods include discriminant function analysis (DFA) and linear discriminant analysis (LDA) which use principal component scores in combination with *a priori* knowledge of the calibration sample identities to aid in the discrimination of classes, i.e., pathogens [35], [36]. DFA and LDA have also proven successful, although primarily with normal Raman analysis of bacteria [15], [19]. More recently a supervised method, partial least squares discriminant analysis (PLS-DA), has been described in which latent variables (LVs) rather than principal components are used for input to build classification models [37], [38]. The advantage of PLS-DA is that LVs are chosen to maximize the variance among different classes, i.e., pathogens, while minimizing the variance within each given class. This approach to spectral analysis gives special emphasis to the vibrational bands that differ among sample types and minimizes the importance of bands that either do not vary among sample types or are inconsistent within the same sample type. A few novel data analysis techniques have also been explored. For example, a spectral barcoding method for bacterial identification based on SERS spectra has been investigated [9], and the use of a quantitative method based on partial least squares (PLS) regression analysis for the identification of several viruses has been used [23].

Previously we have demonstrated SERS-based detection and differentiation of influenza, adenovirus, and respiratory syncytial viruses (RSV) using these OAD substrates [10]. Further studies found that SERS spectra were specific to the strain level, able to detect viruses with gene deletions, and that PLS-DA provided a robust and statistically significant means of rapidly and objectively differentiating each RSV strain [11]. In these previous studies SERS assays were performed with purified viruses in water and buffer. In this report, the specificity and sensitivity of the SERS platform is evaluated for rotaviruses in a biological matrix. Eight tissue culture adapted strains of rotavirus spanning the clinically significant genotypes were analyzed in cell lysates demonstrating the capacity of SERS to detect rotavirus strains in a complex matrix, the capacity to distinguish among strains and genotypes, and the ability to provide a quantitative measurement of the viruses. These studies indicate that the SERS detection method can be used for rotavirus detection, a component central to human health care.

## Materials and Methods

### Rotavirus propagation

Eight laboratory strains representative of the most commonly identified G and P genotypes were propagated in MA104 cells purchased from ATCC (CRL-2378) in the presence of trypsin for SERS-based evaluation, these are detailed in Table 1. Briefly viruses were prepared in MA104 cells grown in DMEM with fetal bovine serum. Virus stocks were activated with 10 µg/ml of porcine trypsin for 30 min at 37°C, and then propagated in MA104 cells in the presence of 1 µg/ml of trypsin. Cells were incubated at 37°C until a cytopathic effect was evident, then lysates were frozen and thawed twice. Hemi-nested RT-PCR assays were employed to confirm the G and P genotype of each rotavirus isolate using type specific primers [39]. The viral tires of all virus stocks were determined by fluorescent focus neutralization assays [40].

**Table 1:** Rotavirus strains and corresponding G and P genotypes.

Strain	G-type	P-type
F45	9	8
RV3	3	6
RV4	1	8
RV5	2	4
S2	2	4
ST-3	4	6
Wa	1	8
YO	3	8

### SERS Substrate preparation

The oblique angle deposition (OAD) of aligned silver nanorod arrays as SERS substrates has been previously described [10]. In brief, 1×1 cm glass microscope slides were cleaned with hot piranha solution (80% sulfuric acid, 20% hydrogen peroxide), and rinsed with deionized water. The substrates were then dried with a stream of N_2_(g) before loading into a custom-designed, high vacuum electron beam evaporation (E-beam) system. Thin films of Ti (20 nm) and Ag (500 nm) were evaporated onto the substrates at a rate of 0.2 nm/s and 0.3 nm/s, respectively, with the incident vapor normal to the substrate surface. The Ti served as an adhesion layer. The substrates were then rotated by 86° with respect to the incident vapor. Ag nanorods were grown at this oblique angle at a rate of 0.3 nm/s until a quartz crystal microbalance (QCM) registered 2000 nm. The QCM was used to monitor the thickness of the film growth in-situ, and was positioned such that it directly faced the incident vapor. As reported elsewhere [10], these deposition conditions result in optimal SERS substrates with overall nanorod lengths of ∼900 nm, diameters of ∼100 nm, densities of ∼13 nanorods/µm^2^, and tilt angle of 71° with respect to the substrate normal.

### SERS measurements

SERS spectra were acquired using a Renishaw inVia confocal Raman microscope system (Hoffman Estates, IL) equipped with a 785 nm near-infrared diode laser as the excitation source. Light from the high power (300 mW) laser was attenuated to ∼7 mW at the sample surface using a series of neutral density filters and focused into a ∼115 µm ×11 µm spot using a 5× objective. SERS spectra were acquired from 400–1800 cm^−1^ in the ExtendedScan mode using three coadded 10 s accumulations. A 1.0-µL aliquot of intact virus was applied to the Ag nanorod array substrate and allowed to evaporate at room temperature prior to spectrum acquisition.

### Data analysis

Preliminary studies were designed to assess the utility of the Ag nanorod substrates to generate SERS spectra of rotaviruses and to evaluate the reproducibility of the method. For these studies, spectra of rotavirus samples were either baseline corrected using a concave rubber band algorithm (OPUS, Bruker Optics, Inc., Billerica, MA) computed with 10 iterations and 64 points or derivatized (1^st^ derivative, 15 point, Savitzky-Golay). These spectral processing steps facilitate visual comparison of the Raman peak positions for spectra collected at different locations on a SERS substrate and for different substrates.

Classification of the rotavirus strains was achieved using partial least squares discriminant analysis that was performed using PLS Toolbox version 4.0 (Eigen Vector Research Inc., Wenatchee, WA), operating in a MATLAB environment (v7.2, The Mathworks Inc., Natick, MA). Multiple PLS-DA models were built to classify the samples according to (1) rotavirus-positive or -negative, (2) strain, (3) G-genotype, or (4) P-genotype. SERS spectra used to generate the PLS-DA classification models were first processed by taking the first derivative of each spectrum (15-point, Savitzky-Golay) and then normalizing to unit vector length [36]. The normalized first derivate spectra were then mean-centered prior to PLS-DA [37], [38]. The same spectral preprocessing protocol was used to generate a quantitative predictive model using partial least squares (PLS) regression analysis.

## Results and Discussion

### SERS detection and reproducibility of Rotavirus samples

At the outset, rotavirus was propagated in MA104 cells and harvested as cell lysates. Virus was diluted to a titer of 10^6^ ffu/mL for SERS evaluation. Initially, a single strain, RV3, was applied to the OAD-fabricated SERS substrate to assess the SERS signal. While SERS bands were detected, the signal was weak, and a thick sample film was observed on the biosensing substrate. SERS is a surface sensitive technique in which only the signal for the viruses in close proximity to the nanorod substrate is enhanced. It is likely that the thick sample layer caused scattering of the laser light prior to reaching the surface thereby significantly reducing signal enhancement. Better signals were be obtained by diluting the rotavirus samples with water, thereby eliminating the sample film and exciting the virus adsorbed directly on the substrate. A 1∶10 dilution of the cell lysate was found to provide an optimum balance of minimizing detrimental multilayer effects and maximizing the sample concentration for increased sensitivity.

**Figure 1:**
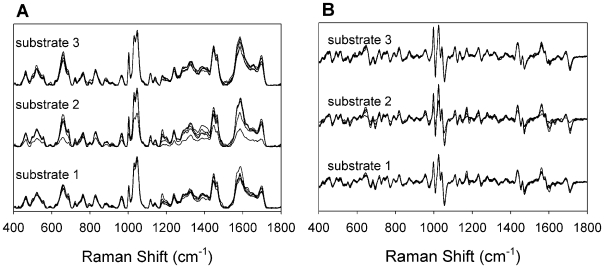
SERS spectral reproducibility. (A) Baseline-corrected SERS spectra for the RV3 strain of rotavirus. Five spectra were collected from different locations for each SERS substrate. Spectra collected from three different substrates are offset for visual comparison. (B) First derivative spectra for those displayed in (A).

A more extensive study was performed to assess the reproducibility of the SERS measurement for RV3. An RV3 sample was applied to three independently prepared OAD-fabricated SERS substrates and five spectra were collected from different locations on each substrate for a total of 15 spectra. The spectra were baseline corrected using an automated algorithm in the OPUS software and are presented in Figure 1A to highlight spot-to-spot spectral variation collected from a single substrate and to demonstrate substrate-to-substrate variation. Review of the spectra reveals several bands between 400 and 1800 cm^−1^. Many of these bands are characteristic of protein and nucleic acid vibrations. For example, the bands at 1572 and 1447 cm^−1^ can be assigned to protein amide vibrations [30], [33], bands at 1122 and 1045 cm^−1^ can be assigned to carbohydrates [41], and the band at 1073 cm^−1^ can be assigned to a carbon-nitrogen stretch [33]. As evident in Figure 1A, the RV3 spectra are similar with respect to the number and location of each Raman band as well as relative intensities. While some variation is a result of sampling and substrate heterogeneities, a significant portion of the variation is a result of baseline artifacts introduced by the baseline subtraction algorithm. The uniformity among spectra is revealed in Figure 1B in which first derivative spectra are displayed. Derivatization is a common means of objectively performing baseline-correction and improves spectral resolution of overlapping bands. The first derivative spectra presented in Figure 1B demonstrate the within and between substrate spectral reproducibility to establish the rationale for the SERS-based detection platform.

### Differentiation of Rotavirus-positive and Rotavirus-negative samples

**Figure 2:**
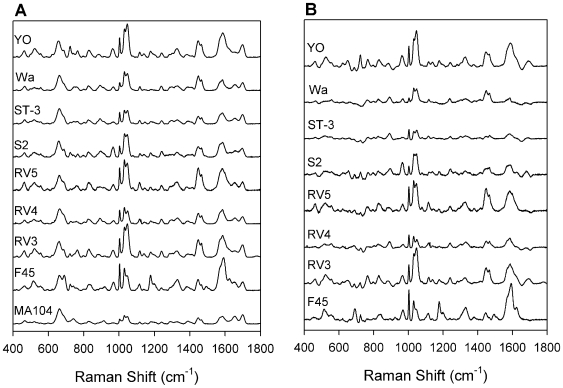
Rotavirus SERS spectra. (A) Average SERS spectra for eight strains of rotavirus and the negative control (MA104 cell lysate). Spectra were baseline corrected, normalized to the band at 633 cm^−1^, and offset for visualization. (B) Difference SERS spectra for eight strains after subtraction of MA104 spectrum.

The SERS spectra for each of the eight rotavirus strains (Table 1) were compared to the spectrum for the uninfected MA104 cell lysate negative control to assess the ability of SERS to differentiate positive from negative samples. Each sample was diluted (1∶10) with water, applied to several OAD-fabricated substrates, and allowed to dry. Average spectra for each rotavirus strain and the negative control collected from three different substrates are shown in Figure 2A. The spectra have been baseline-corrected, normalized to the most intense negative control band (633 cm^−1^), and offset to highlight differences in the relative intensities of each band. The spectra are similar for each strain; however, the relative intensities of each band are different and are a function specific to the strain. It is important to note that the cell lysate produces a SERS spectrum, and given that the rotavirus samples are harvested in a cell lysate, all specimens have this background signal in common. While several matrix bands are common to the rotavirus-positive samples, the overall spectral shape is markedly different between rotavirus-positive and rotavirus-negative samples.

To extract the spectral contribution due to the virus, the MA104 spectrum was subtracted from each rotavirus spectrum. The band at 633 cm^−1^ is the most intense negative control band and is reasonably constant for all samples, thus it is anticipated that this band is due only to a vibrational mode of a matrix component and that changes in band intensity relative to the 633 cm^−1^ band are due to viral vibrations. Therefore, each spectrum was normalized to the band at 633 cm^−1^ prior to spectral subtraction. The difference spectra are plotted in Figure 2B and highlight the bands resulting from rotavirus infection. The results show that each rotavirus sample produces similar SERS spectra with the most notable bands located at 1003, 1030, 1045, and 1592 cm^−1^. The fact that several bands are common to all strains of rotavirus suggests that analysis based on SERS spectral fingerprints can classify samples as rotavirus-positive or rotavirus-negative. We attribute these bands to vibrations of the virus, although it is conceivable that the bands may reflect virus-induced host factors rather than spectra of the virus itself. However, the subsequent sections which accurately categorize the samples according to strain and genotype suggests that the SERS spectra are a direct measurement of the virus particles rather than virus-induced host factors which would not likely classify according to strain and/or genotype. Moreover, we have measured a single virus type propagated in two unique matrices and found consistent peaks among the infected samples (unpublished data), thus providing further evidence that the SERS spectra are a direct measurement of the virus rather than host-response factors.

**Figure 3:**
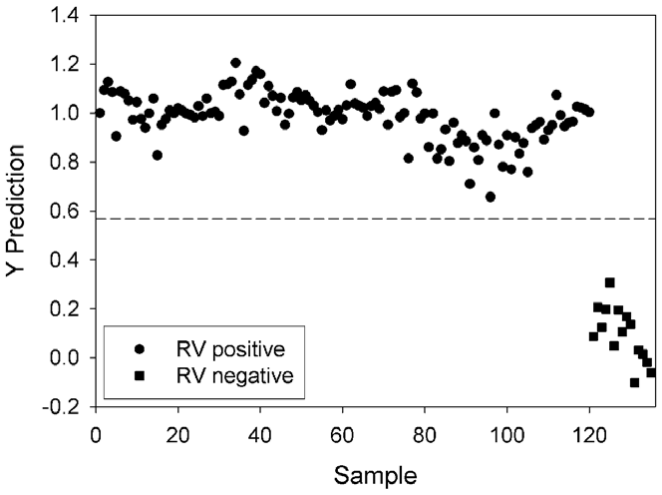
Detection of rotavirus via PLS-DA of SERS spectra. Cross-validation predictions for classification of rotavirus positive and negative samples. Samples which lie above the dashed threshold line are identified as rotavirus positive while those samples which fall below the threshold are identified as negative for rotavirus. True sample identities are given in the legend.

PLS-DA was used to establish statistically significant differences between SERS spectra for rotavirus-positive and rotavirus-negative samples. PLS-DA is a multivariate, full-spectrum calibration method that determines the best-fit mathematical relationship between a descriptor matix, i.e., sample spectra, and a class matrix, i.e., sample identities [37], [38]. PLS-DA is a supervised classification method in which *a prior* knowledge is required for a training dataset in order to build a classification model to test unknown samples. The advantage of this method is in its ability to minimize the contribution of spectral features which vary within a particular sample type and maximize the contribution of spectral features which vary among sample types. Fifteen SERS spectra were collected from three substrates for each of the eight rotavirus-positive samples and the mock-infected MA104 cell lysate negative control to serve as multivariate biological fingerprints. The spectra were vector normalized (see Materials and Methods) to ensure minimal error that is potentially introduced via normalization to an arbitrarily chosen band, e.g., 633 cm^−1^. Each spectrum was assigned to one of two defined classes, rotavirus-positive or rotavirus-negative. A PLS-DA model was built using cross validation (Venetian blinds, 10 splits). Effectively this procedure builds a classification model with 90% of the spectra and then tests the remaining 10% to assess classification accuracy. The process is performed iteratively, for a total of 10 iterations, until each sample is withheld from the model and tested as an unknown. The cross-validated predictions for each collected spectrum are plotted in Figure 3. In Figure 3, each data point is representative of a single SERS spectrum. The PLS Toolbox software generates an optimum threshold for sample classification that is plotted as the dashed line. Spectra which result in Y prediction values greater than the threshold value are classified as rotavirus-positive while those that result in predicted values below the threshold are classified as rotavirus-negative. The optimum rank was selected to minimize classification error of the cross-validated samples and was determined to be 3 latent variables. The true identities of the samples are given in the figure legend and the results indicate that the PLS-DA model correctly classifies each spectrum with 100% accuracy.

### Differentiation of Rotavirus genotypes and Rotavirus strains

As described above, notable differences in the relative intensity of the SERS bands are observed for each rotavirus sample. Given the extreme surface sensitivity inherent to SERS, differences in the relative intensities of each of these bands likely reflect the structural differences in capsid proteins presented to the SERS substrate and reflect antigenic variation, i.e., genotypes. SERS bands from the two major outer capsid proteins, VP7 (G-type) and VP4 (P-type), are expected to dominate the SERS spectra. Based on this premise, it is expected that spectral features resulting from VP7 proteins will be consistent among those viruses belonging to the same G-type. Likewise, it is anticipated that spectral features resulting from VP4 proteins will be consistent among those viruses belonging to the same P-type.

The same spectra evaluated above for the detection of rotavirus were analyzed to assess the ability of SERS to classify the samples according to virus subtypes, i.e., genotypes. First, a PLS-DA model was generated with a classification matrix assigning each spectrum to its specific P-genotype. For example, spectra collected for strains F45, RV4, WA, and YO were assigned to the same class, i.e., P[8], strains RV5 and S2 were assigned to P[4], strains RV3 and ST-3 were assigned to P[6], and one class was reserved for the negative control samples. The PLS-DA model was optimized (8 latent variables) using cross validation (Venetian blinds, 10 splits). P-type class prediction plots for the cross-validated samples are provided as Supporting Information (Figure S1, Supporting Information). The performance of the classification model was evaluated in terms of sensitivity and specificity, where sensitivity is defined as the number of samples assigned to the class divided by the actual number of samples belong to the class, and specificity is defined as the number of samples not assigned to the class defined by the actual number of samples not belonging to the class. Effectively, sensitivity is a measure of false negative results whereas specificity is a measure of false positive results. A summary of the sensitivity and specificity results for the P-type classification model are presented in Table 2. Remarkably, the model resulted in >98% sensitivity and 100% specificity. Only a single spectrum collected for strain Wa (P[8]) was misclassified. Interestingly, this spectrum was not assigned to any of the defined P-types nor was it identified as a negative sample. Although it is desirable for the model to correctly classify the spectrum, it is important to note that the model determined this spectrum to be “unknown” (i.e. not defined by the given classes), and this is preferred rather than mistakenly assigning it to the wrong class, e.g. the negative control.

**Table 2:** Summary of the PLS-DA cross-validation results for classification according to three different models based on the strain, G genotype and P genotype.

P genotype Classification									
	P8	P4	P6	Neg ctrl					
sensitivity	0.983	1.000	1.000	1.000					
specificity	1.000	1.000	1.000	1.000					

G genotype Classification									
	G9	G3	G1	G2	G4	neg ctrl			
sensitivity	1.000	1.000	0.967	1.000	1.000	1.000			
specificity	0.992	1.000	0.990	1.000	1.000	1.000			

Strain Classification									
	F45	RV3	RV4	RV5	S2	ST-3	Wa	YO	neg ctrl
sensitivity	1.000	1.000	1.000	1.000	1.000	1.000	1.000	1.000	1.000
specificity	0.992	1.000	1.000	1.000	1.000	0.992	1.000	1.000	1.000

An additional independent PLS-DA model was built to classify the SERS spectra according to the rotavirus G-type. The rotavirus strains analyzed represented genotypes G1-G4 and G9; thus, these five types, in addition to a negative control group, were defined as classes in the PLS-DA model. A cross-validated model was generated with nine latent variables (Figure S2, Supporting Information) and the model performance is summarized in Table 2. The model resulted in >96% sensitivity and 99% specificity. The slight decrease in performance compared to the P-type model is likely due to fewer representative samples belonging to each G-type. As a result, fewer samples are available for the PLS-DA algorithm to define spectral variation among classes. It is hypothesized that inclusion of more isolates for each of the G-types would result in a more robust classification model.

The definitive achievement in viral identification is specificity at the strain level. A final PLS-DA model was built to differentiate each rotavirus strain based on unique intrinsic SERS spectra (Figure S3, Supporting Information). Each rotavirus strain given in Table 1 and a negative control were defined as unique classes in a PLS-DA model. A rank of 12 resulted in the lowest classification error for cross validated samples, and the performance of the classification model is summarized in Table 2. The strain classification model was 100% sensitive and >99% specific. The results demonstrate the discriminatory power of SERS-based molecular fingerprinting for sample identification. However, it is important to note that only a single virus specimen, i.e., isolated from one subject, for each strain was available for testing. Ideally, several samples of each strain isolated from different subjects would be tested to validate that classification is based on differences in the rotavirus strain. Prospective studies evaluating a greater number of rotavirus-infected subjects are planned, but absence of these more extensive studies does not distract from the important findings shown here.

### Quantitative Analysis of Rotavirus

Partial least squares (PLS) regression analysis was used to explore the quantitative capability of SERS-based molecular fingerprinting. Two concentration ranges were investigated, one dataset covered a higher virus concentration range of 10^5^–10^6^ ffu/mL, and another dataset spanned three orders of magnitude from 10^3^–10^6^ ffu/mL. Dilutions of the rotavirus sample were prepared using the negative control MA104 cell lysate as the diluent to match the background matrix of the virus sample. Water and buffer were not used for dilution since either of these diluents would have altered the lysate matrix concentration, to falsely influence the PLS regression model.

**Figure 4:**
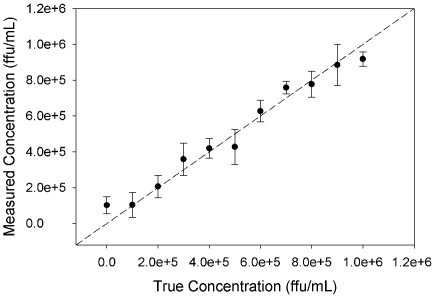
Quantification of high titer rotavirus via SERS. PLS results for analysis of high rotavirus concentration range. Cross-validation predictions of concentration for rotavirus strain ST-3. Dashed line is a plot of x = y to serve as a guide for perfect predictions.

Rotavirus strain ST-3 was selected to assess the high concentration range. Ten calibration samples were prepared between 10^5^ and 10^6^ ffu/mL and SERS spectra were acquired for each concentration. The root mean square error for cross-validation (RMSECV) was analyzed to determine the optimum number of latent variable to include in the PLS model. The RMSECV decreases with the inclusion of each additional initial factor, reaching a minimum value with seven latent variables. Inclusion of additional factors increases the RMSECV due to overfitting of the data. A plot of the predicted rotavirus concentration for cross-validated samples versus the true concentration is presented in Figure 4. Each data point represents the average predicted value and the error bars represent the standard deviation. The plot demonstrates the quantitative accuracy of SERS fingerprinting in combination with chemometric analysis for a small range of relatively high viral titers.

**Figure 5:**
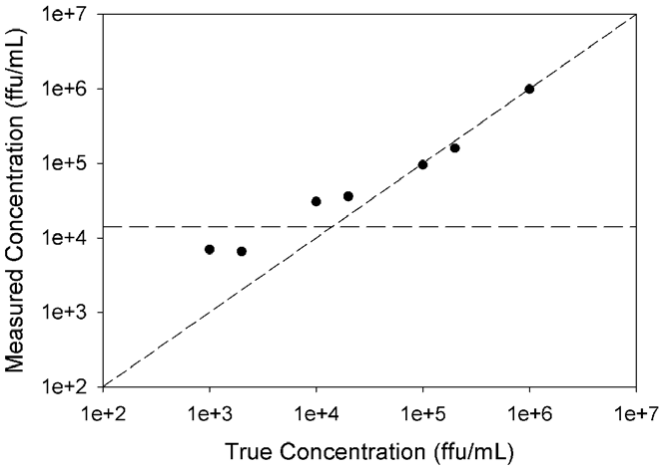
Quantification of low titer rotavirus. PLS results for analysis of extended rotavirus concentration range. Cross-validation predictions of concentration for rotavirus strain YO. Horizontal long dashed line represents the measured rotavirus concentration according to the PLS model for a negative control sample, i.e., cell lysate. Short dashed line is a plot of x = y to serve as a guide for perfect predictions.

After conducting the high concentration range, rotavirus strain YO was selected to assess a more extensive concentration range. Seven test samples were prepared spanning the concentration range of 10^3^–10^6^ ffu/mL. SERS spectra were collected for each concentration and a PLS model was built. The RMSECV was minimized and the quantitative model was optimized with six latent variables. The predicted rotavirus concentration for cross-validated samples versus the true concentration is presented as a logarithmic plot in Figure 5. As is evident, the PLS predicted concentrations based on intrinsic SERS spectra are accurate for concentrations ≥10^4^ ffu/mL. The plot positively deviates at concentrations lower than 10^4^ ffu/mL and the predicted concentrations are elevated with respect to the actual sample concentration. The predicted rotavirus concentration for the negative lysate control is 1.4×10^4^ ffu/mL. Complications from the complex background matrix caused the poor predictive value at these lower titers, and spectral features due to the cell lysate dominate the SERS signature.

### Conclusions

There is an unmet need for the development of a rapid, sensitive test for the identification of viruses and classification of viral strains. The development of a SERS-based biosensor and its application to the rapid detection and differentiation of rotavirus genotypes and rotavirus strains is presented. The OAD fabrication method is capable of producing robust, reproducible biosensing SERS substrates which provide extremely high enhancement factors. Virus samples supplied in a biological matrix were directly applied to the OAD prepared substrates without pretreatment and a SERS viral fingerprint was collected in 30 s. Chemometric methods of data analysis, such as PLS-DA, facilitated the classification of the virus samples based on spectral differences. Four classification models based on different criteria are presented in which >96% of the samples were correctly classified in each model. Moreover, a quantitative model based on PLS resulted in a detection limit of ∼10^4^ ffu/mL for cell lysate preparations of rotavirus. To date only two previous studies have addressed quantitative detection of viruses via intrinsic SERS fingerprinting. The first of these studies was conducted in our laboratory for the detection of respiratory syncytial virus (RSV) [10]. That work relied on univariate, i.e., single wavenumber, analysis of an RSV band resulting in a detection limit of 10^2^ pfu/mL. An important difference between our previous and current work is the sample type that was analyzed. The RSV samples were purified in water whereas the rotavirus samples were suspended in a complex cellular matrix. In a recent report, a commercial off-the-shelf SERS substrate was employed to detect and quantify bovine papular stomatitis virus, pseudocowpox virus, and Yaba monkey tumor virus [23]. Each of these viruses was purified and suspended in deionized water. Consistent with our results for purified virus, the detection limits for these purified viruses were found to be 10^2^ pfu/mL. The apparent decrease in sensitivity for the rotavirus samples in this study is attributed to interference in the SERS signal due to the background matrix medium. While the detection limit for rotavirus in a biological matrix is less sensitive than those reported limits for purified virus samples, this report is a first assessment of biological samples and aids in identifying challenges associated with the current protocol to direct future research to unlock the potential of SERS-based detection. It is important to note that in addition to affecting the detection limit, the sample matrix significantly impacts the classification model. For example, a classification model built with training samples from cell lysate can only be used accurately to classify unknown cell lysate samples. With the current methodology, not only is a calibration model necessary for each virus, but a model is also needed for each sample type, i.e., cell lysate, nasal wash, fecal, etc. Moreover, donor and/or day-to-day variations in these complex clinical specimens will cause the signal to fluctuate due to the matrix itself. Nonetheless, this study demonstrates the power of SERS to differentiate individual strains of viruses in less than one minute when coupled to chemometric methods for data analysis, and clearly demonstrates the tremendous advantage of intrinsic SERS-based detection of viruses compared to more traditional methods with respect to the detection speed and the ease of genotyping.

The data also define the critical next steps in pursuit of a robust method of viral fingerprinting, namely, overcoming the challenges imposed by a complex and dynamic sample matrix. There are three approaches that can potentially address these challenges by discriminating against the matrix itself, or its signal, in favor of the virus or virus signal. First, there is the potential to include a simple sample filtration step to isolate the virus thereby removing the background matrix from the analysis. Commercial filters are currently available from Millipore for isolating adenovirus and lentivirus, with potential application to other viruses. Second, strategies to increase sensor selectivity can be explored. More traditional recognition elements such as an antibody or aptamer are potential candidates, although they may not be effective for emerging or mutant isolates. Alternatively, more novel perm-selective barriers are being explored for species selectivity [42]. With this approach only the virus binds to the SERS substrate to produce a detectable signal and the background matrix is removed via washing; in effect, the signal will be independent of the matrix. Third, advances in chemometric methods can potentially aid in the enhanced selection of virus signal in the presence of a complex background. For example, methods can potentially be developed to select SERS bands that are consistent for a particular virus across a number of clinical specimens and to identify those SERS bands which vary due to matrix fluctuation to build a more robust classification model. It has been suggested that second-order multivariate calibration methods can accurately detect the analyte in the presence of unknown, i.e., unmodeled, interferences [23]. Experiments are currently underway to investigate each of these approaches for improved detection.

### Supporting Information Available

Additional information as noted in text. This material is available free of charge via the Internet.

## Supporting Information

Figure S1 Cross validation results for PLS-DA P genotype classification of RV samples and negative control based on SERS spectra. P8 (green circles, F45, RV4, WA, YO), P6 (blue crosses, RV3, ST-3), P4 (red triangles, RV5, S2), (black circles, negative control).
(0.67 MB TIF)

Figure S2 Cross validation results for PLS-DA G genotype classification of RV samples and negative control based on SERS spectra. G9 (blue triangles, F45), G3 (blue squares, RV3, YO), G1 (red triangles, RV4, WA), G2 (green crosses, RV5, S2), G4 (blue crosses, ST-3), negative control (black circles).
(0.89 MB TIF)

Figure S3 Cross validation results for PLS-DA strain classification of RV samples and negative control based on SERS spectra.
(1.30 MB TIF)
