# Supplementary figures and images for: Rapid and Sensitive Detection of Rotavirus Molecular Signatures Using Surface Enhanced Raman Spectroscopy
Source: PLoS One. 2010 Apr 19;5(4):e10222. doi: 10.1371/journal.pone.0010222 (PMC2856680; doi:10.1371/journal.pone.0010222)

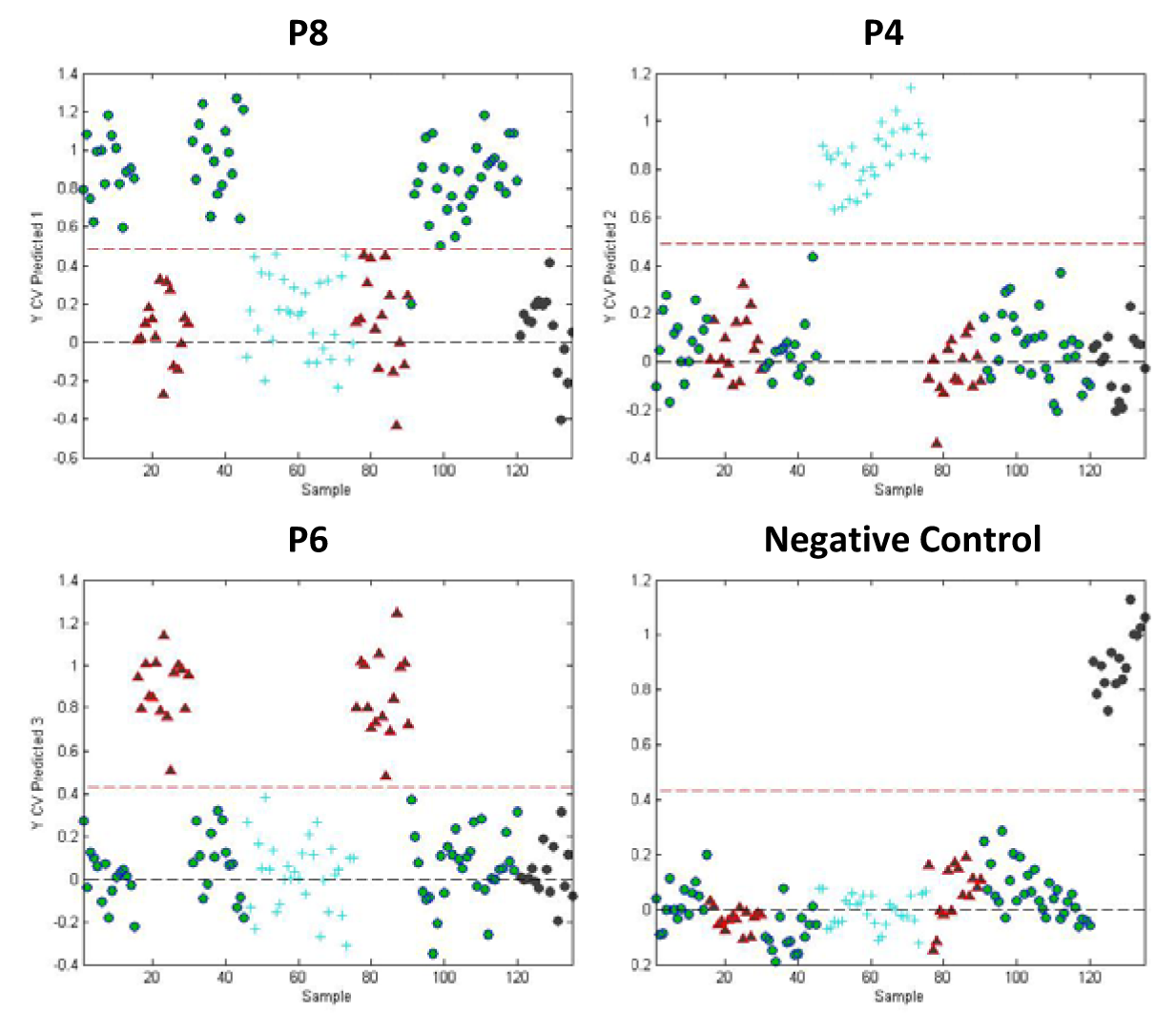

Supplement: Figure S1 — Cross validation results for PLS-DA P genotype classification of RV samples and negative control based on SERS spectra. P8 (green circles, F45, RV4, WA, YO), P6 (blue crosses, RV3, ST-3), P4 (red triangles, RV5, S2), (black circles, negative control). (0.67 MB TIF) [file pone.0010222.s001.tif]

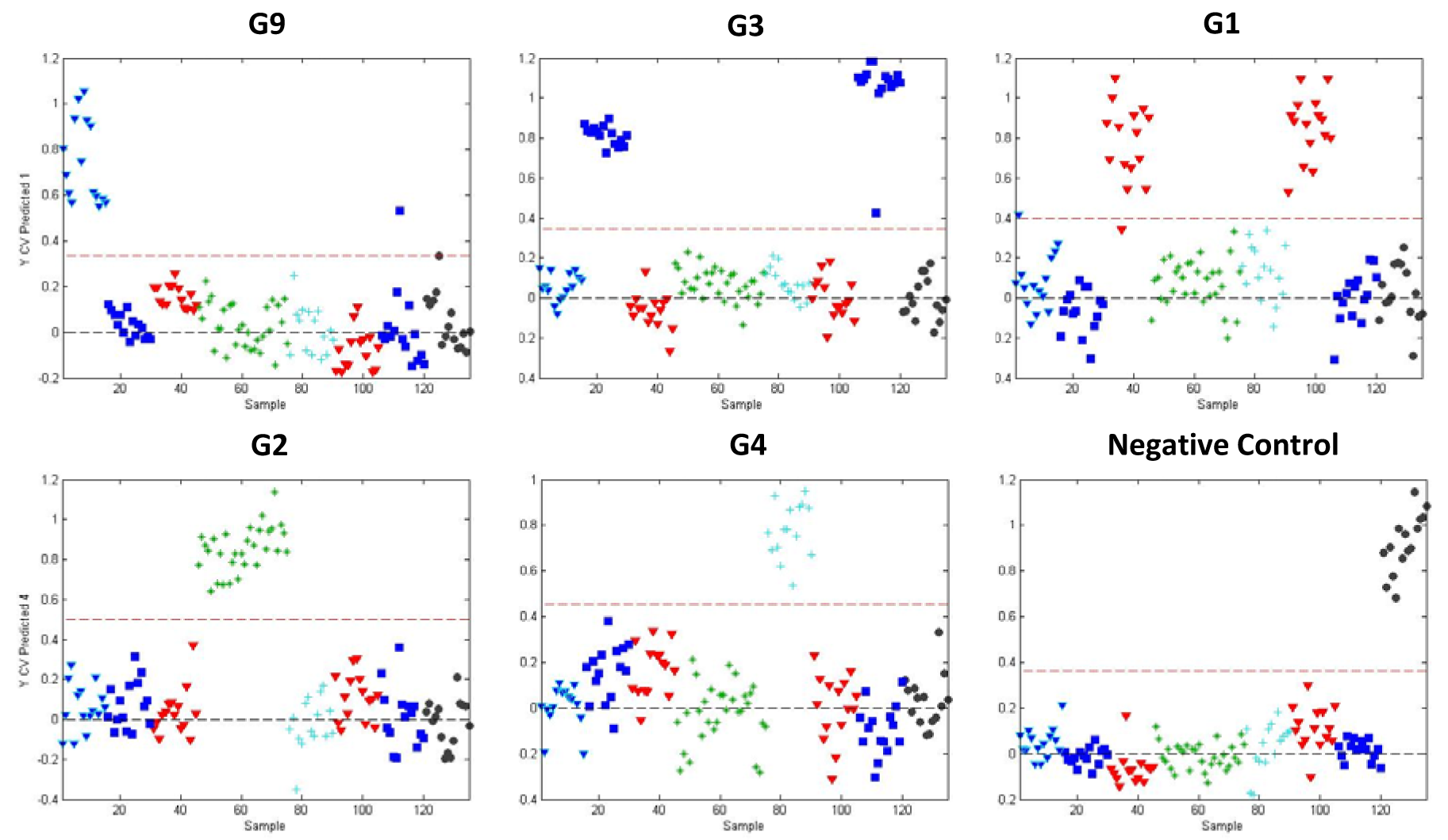

Supplement: Figure S2 — Cross validation results for PLS-DA G genotype classification of RV samples and negative control based on SERS spectra. G9 (blue triangles, F45), G3 (blue squares, RV3, YO), G1 (red triangles, RV4, WA), G2 (green crosses, RV5, S2), G4 (blue crosses, ST-3), negative control (black circles). (0.89 MB TIF) [file pone.0010222.s002.tif]

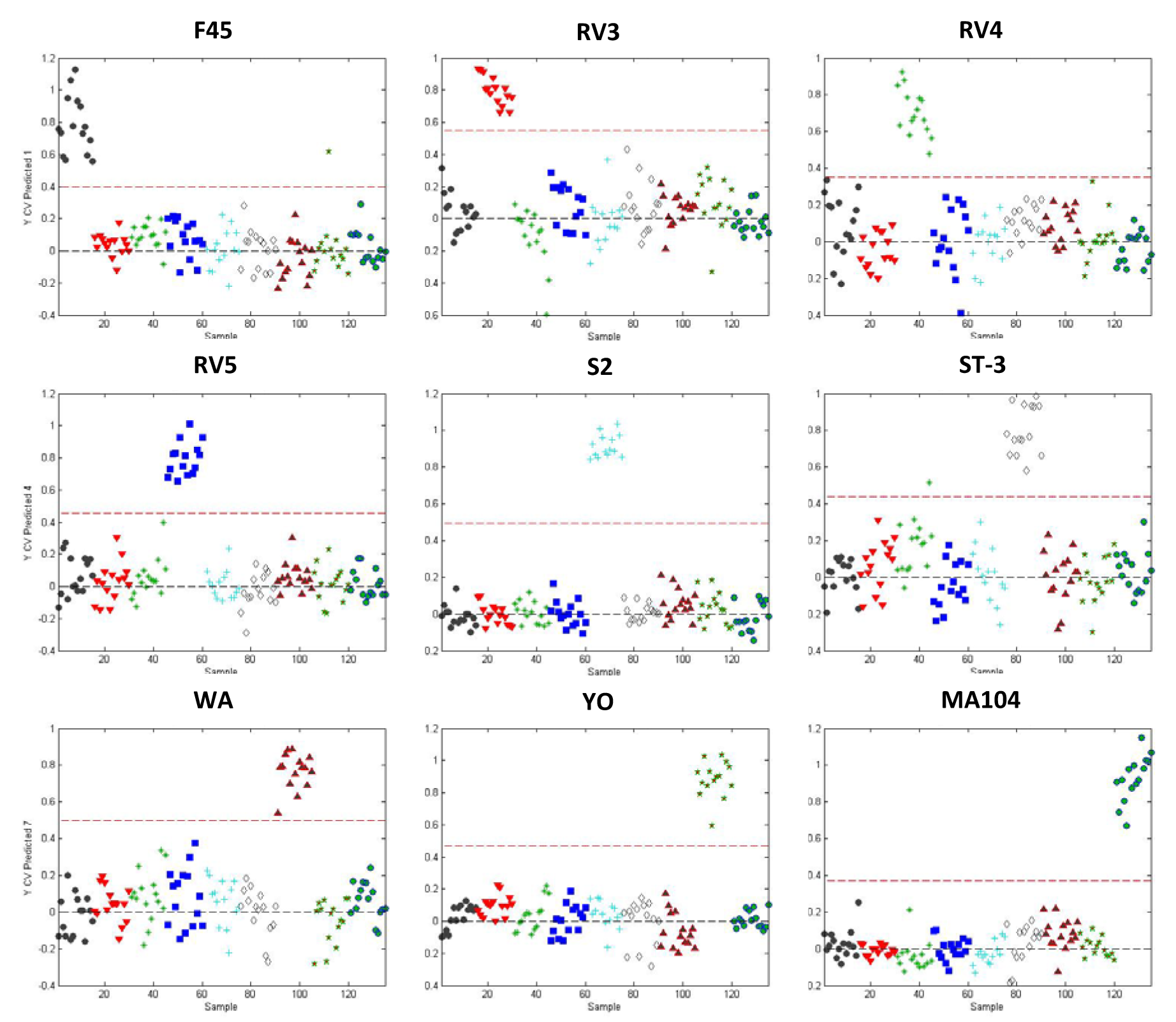

Supplement: Figure S3 — Cross validation results for PLS-DA strain classification of RV samples and negative control based on SERS spectra. (1.30 MB TIF) [file pone.0010222.s003.tif]
